# Venous thromboembolism risk following surgery during the COVID‐19 pandemic

**DOI:** 10.1111/anae.70091

**Published:** 2025-12-03

**Authors:** Andrew Jackson, David J. Humes, Amir Mehrkar, Sebastian C. J. Bacon, Simon Davy, Ben Goldacre, Joe West, Colin J. Crooks

**Affiliations:** ^1^ Gastrointestinal and Liver Theme, National Institute for Health Research Nottingham Biomedical Research Centre, Nottingham University Hospitals NHS Trust Nottingham UK; ^2^ University of Nottingham, School of Medicine, Queen's Medical Centre Nottingham UK; ^3^ Bennett Institute for Applied Data Science, Nuffield Department of Primary Care Health Sciences University of Oxford Oxford UK; ^4^ Lifespan and Population Health, University of Nottingham, School of Medicine Nottingham UK

**Keywords:** COVID‐19, surgery, vaccine, venous thromboembolism

## Abstract

**Introduction:**

SARS‐CoV‐2 infection is associated with an increased risk of venous thromboembolism. Data are lacking on how this risk altered during the COVID‐19 pandemic and following vaccination. We aimed to evaluate the 90‐day risk of postoperative venous thromboembolism during the pandemic.

**Methods:**

We performed a retrospective cohort study of patients having abdominal, obstetric, orthopaedic, cardiac, thoracic or vascular surgical procedures using the OpenSAFELY‐TPP platform. Crude 90‐day risks of venous thromboembolism were calculated and crude and adjusted hazard ratios were derived from individual Cox proportional hazards models.

**Results:**

In total, 1,800,540 procedures were performed with 15,390 individual venous thromboembolic events recorded within 90 days. The highest crude absolute risk was in the Alpha wave at 1.2%. Postoperative SARS‐CoV‐2 infection was associated with a 4.4‐fold increase in relative risk of 90‐day venous thromboembolism (adjusted hazard ratio 4.42, 95%CI 4.21–4.64) compared with those without. Recent SARS‐CoV‐2 infection was associated with an increased risk of venous thromboembolism (adjusted hazard ratio 4.03, 95%CI 3.78–4.30) compared with those without. Patients who were unvaccinated had the highest relative risk for 90‐day venous thromboembolism. A single dose of vaccine was associated with a 20% relative risk reduction of venous thromboembolism (adjusted hazard ratio 0.80, 95%CI 0.76–0.84).

**Discussion:**

SARS‐CoV‐2 infection status and vaccination history were associated with 90‐day venous thromboembolism risk, with both recent and postoperative SARS‐CoV‐2 infection associated with an increased risk, whilst one dose of vaccine reduced the risk.

## Plain Language Summary

People who get COVID‐19 (caused by the SARS‐CoV‐2 virus) are more likely to develop blood clots in their veins. These clots are called venous thromboembolism (VTE). Doctors wanted to know how this risk changed during the COVID‐19 pandemic and after people started getting vaccinated. The goal of this study was to find out how often people had blood clots within 90 days after surgery during the pandemic. The researchers looked back at medical records of people who had certain types of surgery, such as abdominal, obstetric, bone (orthopaedic), heart, chest, or blood vessel operations. They used a large database called OpenSAFELY‐TPP. They counted how many people had blood clots within 90 days of surgery and compared different groups of patients, including those who had COVID‐19 and those who did not. The study included about 1.8 million surgeries, and 15,390 people had a blood clot within 90 days. The highest risk of clots happened during the Alpha wave of COVID‐19, when about 1 in 100 patients (1.2%) developed a clot. People who caught COVID‐19 after surgery were about 4 times more likely to get a blood clot than those who didn't. People who had recent COVID‐19 (before surgery) were also at higher risk. Those who were not vaccinated had the highest risk of clots. Having one vaccine dose lowered the risk by about 20%. Getting COVID‐19, either before or after surgery, made blood clots more likely within 90 days. However, getting at least one dose of the vaccine helped reduce that risk.

## Introduction

Venous thromboembolism (VTE), encompassing deep vein thrombosis and pulmonary embolism, affects nearly 10 million people each year globally and is a leading cause of preventable death in hospitals in high‐income countries [1, 2]. The risk factors associated with postoperative VTE are multifaceted and include patient‐specific characteristics; severity of surgical insult; and peri‐operative management. The COVID‐19 pandemic introduced a new dimension to our understanding of VTE susceptibility during the postoperative period.

In addition to the respiratory symptoms induced by SARS‐CoV‐2, early evidence suggested that the virus had the capacity to induce a hypercoagulable state, resulting in an increased incidence of thrombotic events among infected patients [3, 4, 5]. The COVIDSurg Collaborative assessed the risk of a postoperative SARS‐CoV‐2 infection, reporting a 1.5‐fold increase in the risk of VTE (adjusted odds ratio (aOR) 1.5 95%CI 1.1–2.0) and an increased 30‐day mortality rate [6]. SARS‐CoV‐2 infection before surgery was also associated with an increased VTE risk (aOR 1.9, 95%CI 1.2–3.3). However, this study was conducted early in the COVID‐19 pandemic, and subsequent SARS‐CoV‐2 variants and vaccinations are likely to have changed the associated risk of VTE following surgery.

Currently, guidelines based on the COVIDSurg study remain in place to consider delaying surgery for patients with recent SARS‐CoV‐2 infection for 2 weeks, and for those who are not having low‐risk surgery or are not at low risk, an individual risk assessment is advised to balance the risk of benefit from delay vs. harm [7]. A recent systematic review has highlighted the variation in VTE incidence during the COVID‐19 pandemic following surgery, with increased rates after orthopaedic; emergency general; and gastrointestinal surgery [8]. However, the review highlighted that the included studies did not consider the impact of later stages of the pandemic, a wide range of surgical types or the impact of vaccination on VTE risk [8]. A recent population‐based study using OpenSAFELY data included pulmonary complications but not a detailed analysis of the risk of VTE [9]. Given that COVID‐19 is now endemic and remains a global public health threat, it is important to understand how the risk of SARS‐CoV‐2 infection affects the risk of VTE following surgery to avoid unnecessary delays. We therefore did a population‐based study of the risk of VTE following SARS‐CoV‐2 infection, accounting for SARS‐CoV‐2 variant and vaccination status.

## Methods

Following ethical approval, we did a retrospective population‐based cohort study aiming to evaluate the 90‐day risk of postoperative VTE in adults during the COVID‐19 pandemic between 1 February 2020 and 1 October 2022. We used routinely collected electronic health record data from English primary care practices managed by the software provider TPP (Horsforth, UK) through OpenSAFELY, covering approximately 40% of the population in England. All data were linked, stored and analysed securely using the OpenSAFELY platform as part of the NHS England OpenSAFELY COVID‐19 service. The dataset is based on 24 million people registered with primary care practices using TPP SystmOne software. Primary care data were linked through OpenSAFELY to: inpatient hospital records via the hospital episode statistics from NHS Digital; national coronavirus testing records from the second‐generation surveillance system; Office for National Statistics death data; and vaccination records from the National Immunisation Management System. Data were pseudonymised and included coded diagnoses, medications and physiological parameters, with no free text data included.

All adults aged ≥ 18 y who were registered with a primary care practice linked to OpenSAFELY‐TPP in England and underwent a surgical procedure associated with a hospital admission were identified using OPCS‐4 codes from Hospital Episode Statistics data. These procedures were then grouped into broad categories and defined using code lists (opencodelists.org), with specific code lists used in this study available at github.com/opensafely/PostOpCovid/tree/main/codelists. The analysis allowed for the inclusion of up to five procedures per patient. Patients were followed up from surgery admission date until they developed a VTE event; deregistered from a participating GP; died; or 90 days following the index admission.

Patient age at the time of surgery was categorised into the following groups: 18–50 y; 51–70 y; 71–80 y; and ≥ 80 y. These age bands were selected to allow a balance of VTE events between groups to allow sufficient power for the planned stratified analysis. Sex (male and female) and geographic regions were categorised according to commonly applied systems. Socio‐economic status was defined using the 2019 English Index of Multiple Deprivation Scores, which were divided into quintiles [10]. Surgical procedures were categorised into abdominal (gastrointestinal, urological and gynaecological); obstetric; orthopaedic; and a combined category of cardiac, thoracic and vascular (including endovascular) procedures. In addition, procedures were classified as major or intermediate/minor based on standard definitions [11], and defined as elective or emergency according to the recorded admission classification. For each patient, the first procedure for each category or procedure type was selected for the analysis. The underlying diagnosis was classified as benign or malignant according to ICD‐10 codes recorded at discharge. The BMI was categorised into four groups: underweight (≤ 18 kg.m^‐2^); normal (18–24 kg.m^‐2^); overweight (24–29 kg.m^‐2^); and obese (> 29 kg.m^‐2^). Comorbidities were identified through primary care codes before admission and categorised using the Charlson comorbidity index [12]: 0 for no comorbidity; 1 for a single comorbidity; and 2 for multiple or severe comorbidities.

Timing of admission date during COVID‐19 was used to stratify the analysis, with the pandemic periods divided according to the dominant viral variants, as defined by the Office for National Statistics [13]. These were the pre‐Alpha variant (wave 1) from 1 February 2020 to 7 December 2020; Alpha variant (wave 2) from 8 December 2020 to 17 May 2021; Delta variant (wave 3) from 18 May 2021 to 19 December 2021; and Omicron variant (wave 4) from 20 December 2021 until the end of the study period.

Exposure to SARS‐CoV‐2 was categorised according to its temporal relationship with the surgical procedure. Positive tests that occurred within 90 days of a previous positive test were ignored as the test could remain positive for up to 90 days. Recent SARS‐CoV‐2 infection was defined as a positive SARS‐CoV‐2 test occurring between 42 days and 8 days before the admission date. Previous SARS‐CoV‐2 infection was defined as a positive test occurring more than 42 days before the admission date. Postoperative and current SARS‐CoV‐2 infection was identified if a positive SARS‐CoV‐2 test occurred 7 days before the procedure or during the 90‐day follow‐up period. COVID‐19 vaccination status was categorised based on the number of vaccine doses received by the admission date and was classified as unvaccinated or as having received one dose, two doses or three doses (booster).

Primary outcome was the occurrence of a VTE event (deep vein thrombosis or pulmonary embolism) within 90 days following admission for a surgical procedure. Through the OpenSAFELY‐TPP platform, VTE events were identified using ICD‐10 codes from Hospital Episode Statistics data and corresponding Read codes within GP records. Each identified event was validated if corroborated by a prescription for an anticoagulant medication included in the British National Formulary and licenced for the treatment of VTE, occurring between 15 days before and 90 days after a VTE diagnosis. The use of primary care data to validate a VTE diagnosis has been described and used previously [14, 15, 16, 17].

The primary analysis aimed to provide overall 90‐day VTE risk estimates following admission for a surgical procedure for the clinical and demographic covariates described previously. For each covariate strata, the number of patients at risk and the number of postoperative VTE events within 90 days were determined as counts rounded to the nearest 10. The crude 90‐day cumulative risk of VTE within each stratum was estimated using the Kaplan–Meier method, accounting for censoring. Crude hazard ratios (HRs) for VTE were derived from individual Cox proportional hazards models for each covariate to assess relative risk. To control for potential confounding factors, adjusted HRs (aHRs) were obtained from a fully adjusted Cox model that included all covariates simultaneously. Both crude HRs and aHRs are presented with 95%CIs.

To explore changes in postoperative VTE risk across the waves of the pandemic and the effects of vaccination, a subgroup analysis was performed on a high‐risk surgical population undergoing major elective surgery. This cohort was selected as it represented a controlled population, with established clinical guidelines for thromboprophylaxis and the potential for targeted VTE prevention strategies. Cox proportional hazards models were fitted to estimate the 90‐day VTE risk following an admission for surgery and adjusted for age; sex; vaccination status; current cancer diagnosis; and Charlson comorbidity index. COVID‐19 waves were included as covariates within the model, with interaction terms between each of the waves and broad procedure types (abdominal, orthopaedic, obstetric, cardiothoracic and vascular). Interactions were also included between waves and patient SARS‐CoV‐2 infection status (recent, postoperative or previous). The 90‐day VTE risk estimates were calculated and adjusted for the competing risk of readmission and death. To show how the predicted adjusted VTE risk varied with different combinations of predictors, VTE risks were calculated for differing scenarios according to vaccination status, procedure type and timing of SARS‐CoV‐2 infection with an exemplar 50–70‐year‐old female patient with a normal BMI (20 kg.m^‐2^), a single comorbidity, current cancer diagnosis, from the East Midlands, residing in an area ranked in the third quintile for deprivation.

Data management was performed using Python version 3.8, with analysis carried out using R version 4.0 (R Foundation for Statistical Computing, Vienna, Austria).

## Results

A total of 1,800,540 surgical procedures were performed, with 15,390 individual VTE events recorded during the 90‐day follow‐up period. In total, 23,420 patients (1.3%) had a SARS‐CoV‐2 infection in the 90 days after surgery; 12,030 (0.7%) had a recent SARS‐CoV‐2 infection; and 64,260 (3.6%) had a previous SARS‐CoV‐2 infection (Table 1).

**Table 1:** Cumulative incidence of postoperative venous thromboembolism (VTE) risk for the study population and crude and adjusted hazard ratios for the associated risk.

	Number at risk	90‐day VTE events	Crude 90‐day cumulative VTE risk*	Crude 90‐day hazard ratio (95%CI)	Adjusted 90‐day hazard ratio (95%CI)
Procedure					
Abdominal	587,720	4520	0.77	Ref	
Cardiothoracic and vascular	76,530	1170	1.57	2.12 (1.99–2.27)	1.43 (1.34–1.53)
Obstetrics	536,860	830	0.16	0.19 (0.17–0.20)	1.63 (1.48–1.80)
Orthopaedic	599,430	8870	1.50	1.89 (1.82–1.96)	1.27 (1.21–1.32)
Age; y					
18–50	966,650	2030	0.21	Ref	
51–70	453,870	3830	0.85	4.14 (3.92–4.37)	2.58 (2.40–2.76)
71–80	246,650	3870	1.59	7.94 (7.52–8.37)	3.83 (3.56–4.13)
≥ 81	133,370	5660	4.42	23.18 (22.03–24.39)	6.25 (5.80–6.73)
Sex					
Female	1,209,180	8470	0.71	Ref	
Male	591,370	6930	1.19	1.74 (1.68–1.79)	1.13 (1.09–1.17)
BMI; kg.m^‐2^					
≤ 18	10,320	320	3.28	Ref	
18–24	110,820	1490	1.37	0.41 (0.36–0.46)	0.70 (0.62–0.79)
24–29	150,290	1430	0.97	0.29 (0.25–0.32)	0.56 (0.50–0.64)
29–100	188,440	1670	0.89	0.26 (0.23–0.30)	0.65 (0.57–0.73)
Missing	1,340,670	10,480	0.79	0.23 (0.21–0.26)	0.59 (0.52–0.66)
Index of multiple deprivation quintile					
1	378,270	3100	0.83	Ref	
2	349,450	2960	0.86	1.03 (0.98–1.09)	0.96 (0.91–1.01)
3	373,120	3270	0.89	1.07 (1.02–1.12)	0.91 (0.87–0.96)
4	353,560	3170	0.91	1.09 (1.04–1.15)	0.88 (0.84–0.93)
5	298,880	2690	0.91	1.10 (1.04–1.16)	0.86 (0.81–0.90)
Missing	47,240	190	0.42	0.49 (0.43–0.57)	0.89 (0.77–1.03)
COVID‐19 wave					
1 (pre‐Alpha)	513,100	4860	0.96	Ref	
2 (Alpha)	278,360	2800	1.02	1.07 (1.02–1.12)	1.13 (1.07–1.19)
3 (Delta)	431,770	3220	0.75	0.78 (0.75–0.82)	1.05 (0.99–1.12)
4 (Omicron)	577,310	4510	0.79	0.82 (0.79–0.85)	0.85 (0.81–0.89)
Major surgery					
No	1,061,320	3730	0.35	Ref	
Yes	739,230	11,670	1.60	4.82 (4.64–5.00)	1.64 (1.57–1.72)
Vaccination status					
Unvaccinated	1,171,360	8550	0.80	Ref	
1 dose	175,190	2250	1.08	1.37 (1.31–1.44)	0.80 (0.76–0.84)
2 doses	298,350	2820	0.91	1.16 (1.11–1.21)	0.73 (0.69–0.77)
3 doses	155,630	1770	0.90	1.13 (1.08–1.19)	0.71 (0.66–0.75)
Region					
East	408,150	3630	0.90	Ref	
East Midlands	315,200	2830	0.91	1.02 (0.97–1.07)	0.95 (0.91–1.00)
London	94,030	540	0.59	0.66 (0.60–0.72)	0.82 (0.75–0.90)
North East	90,800	790	0.88	0.99 (0.92–1.07)	0.92 (0.85–0.99)
North West	174,270	1560	0.91	1.02 (0.96–1.08)	0.94 (0.88–0.99)
South East	122,670	1030	0.85	0.95 (0.89–1.02)	0.90 (0.84–0.96)
South West	258,600	2140	0.84	0.94 (0.89–0.99)	0.86 (0.81–0.91)
West Midlands	67,690	590	0.90	1.01 (0.92–1.10)	0.96 (0.88–1.05)
Yorkshire and The Humber	269,110	2250	0.84	0.94 (0.89–0.99)	0.91 (0.87–0.96)
Current cancer diagnosis					
No	1,692,930	13,080	0.78	Ref	
Yes	107,620	2320	2.20	3.01 (2.88–3.15)	2.63 (2.49–2.78)
Admission type					
Elective	1,429,900	5430	0.38	Ref	
Emergency	370,650	9970	2.78	7.95 (7.69–8.22)	4.06 (3.9–4.24)
Charlson comorbidity index score					
None	1,418,550	6060	0.44	Ref	
Single	182,200	2940	1.55	3.75 (3.59–3.92)	1.48 (1.41–1.55)
Multiple or severe	199,800	6400	3.09	7.83 (7.56–8.11)	1.93 (1.85–2.02)
Recent SARS‐CoV‐2 infection					
No	1,788,520	14,380	0.81	Ref	
Yes	12,030	1020	8.95	12.24 (11.48–13.04)	4.03 (3.78–4.30)
Previous SARS‐CoV‐2 infection					
No	1,736,290	14,800	0.86	Ref	
Yes	64,260	600	0.97	1.11 (1.02–1.20)	2.21 (2.04–2.41)
Postoperative SARS‐CoV‐2 infection					
No	1,777,130	13,070	0.76	Ref	
Yes	23,420	2320	5.32	6.41 (6.13–6.71)	4.42 (4.21–4.64)

* Adjusted for censoring.

Each patient is able to undergo up to five procedures.

Totals rounded to 10.

Patients who had cardiac, thoracic or vascular surgery had the highest absolute crude risk of 90‐day VTE (1.6%), representing a 1.4‐fold increase in risk compared with those who had abdominal surgery (aHR 1.43, 95%CI 1.34–1.53). Although patients undergoing obstetric procedures showed the lowest absolute crude VTE risk (0.2%), they had the highest relative risk increase after adjusting for confounders (aHR 1.63, 95%CI 1.48–1.80).

Advanced age was associated with an increased 90‐day VTE risk (Table 1). Malignancy was associated with an increased VTE risk, with patients who had a current cancer diagnosis having a > 2.6‐fold increase in risk (aHR 2.63, 95%CI 2.49–2.78). Similarly, patients with more comorbidities (Charlson comorbidity index ≥ 2) were at increased risk compared with those without (aHR 1.93, 95%CI 1.85–2.02).

There was variation in 90‐day VTE risk seen across the different pandemic waves, with the highest crude absolute risk in the Alpha wave at 1.2%. The risk of postoperative VTE decreased across the waves to its lowest during the Omicron wave (aHR 0.85, 95%CI 0.81–0.89) compared with the pre‐Alpha wave.

Patient SARS‐CoV‐2 infection status had a substantial influence on 90‐day VTE risk. Those who developed a postoperative SARS‐CoV‐2 infection had a 4.4‐fold increase in relative risk of 90‐day VTE (aHR 4.42, 95%CI 4.21–4.64) compared with those who remained infection‐free following surgery. Similarly, patients with a recent SARS‐CoV‐2 infection also showed an increased risk (aHR 4.03, 95%CI 3.78–4.30) compared with those without a recent infection.

Patients who were unvaccinated had the highest relative risk for 90‐day VTE. Following a single dose of the vaccine, there was a 20% relative risk reduction (aHR 0.80, 95%CI 0.76–0.84). This risk fell further after two and three vaccine doses, with relative risk reductions of 27% (aHR 0.73, 95%CI 0.69–0.77) and 29% (aHR 0.71, 95%CI 0.66–0.75), respectively.

**Figure 1:**
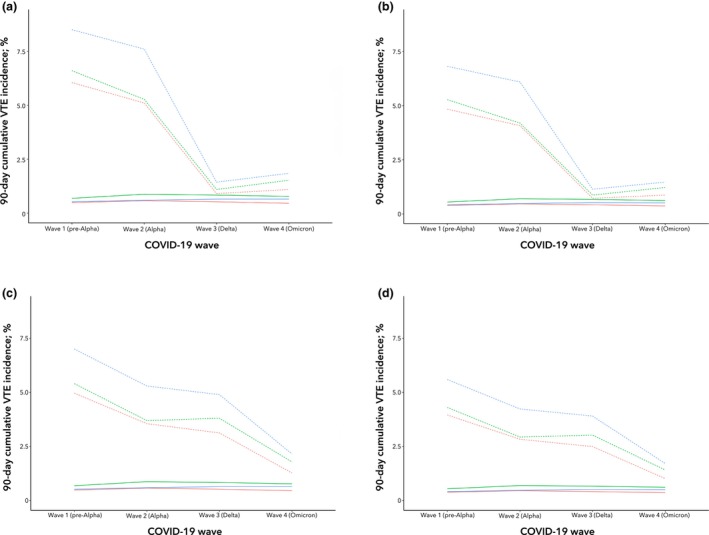
Predicted risk of postoperative venous thromboembolism (VTE) across each wave of the pandemic for each surgical specialty stratified by (a) postoperative COVID‐19 in patients who were unvaccinated; (b) postoperative COVID‐19 in patients who were vaccinated; (c) recent COVID‐19 in patients who were unvaccinated; and (d) recent COVID‐19 in patients who were vaccinated. Orange line, abdominal surgery; green line, cardiothoracic and vascular surgery; blue line, orthopaedic surgery. Dotted lines of the same colours indicate postoperative COVID‐19 infection.

The greatest predicted risk was seen in patients who were unvaccinated and undergoing orthopaedic procedures, and this fell from 8.5% in wave 1 (pre‐Alpha) to 1.9% in wave 4 (Omicron) (Fig. 1). In comparison, for patients who were vaccinated, the risk fell to 1.5% by wave 4 (Omicron) (Fig. 1). For all procedure types, the greatest predicted reduction in risk was seen between wave 2 (Alpha) and wave 3 (Delta) in the postoperative COVID‐19 model. In the Omicron wave, all risks approached baseline.

Across all four waves of the pandemic, there was an increased risk of VTE associated with patients who had a recent pre‐operative SARS‐CoV‐2 infection (Fig. 1). Similar to postoperative SARS‐CoV‐2, this risk was highest during wave 1 (pre‐Alpha) across all major elective surgery categories. The risk was greatest in patients having orthopaedic procedures (7.0%), but this reduced in subsequent waves (Fig. 1). The risk of postoperative VTE in patients who were vaccinated after a recent infection was lower across all waves of the pandemic (Fig. 1).

## Discussion

This study provides a detailed understanding of how the postoperative risk of VTE evolved during the COVID‐19 pandemic across multiple surgical specialties. Postoperative SARS‐CoV‐2 infection was associated with a 4.4‐fold risk of 90‐day VTE compared with those without. Risks were highest at the beginning of the pandemic, with an overall 15% reduction in risk by the Omicron wave across all surgical specialties compared with the original pre‐Alpha wave. COVID‐19 vaccination conferred protection against VTE risk, with each successive vaccine dose offering a stepwise reduction. Patients who were fully vaccinated experienced a 29% reduction in relative risk of VTE compared with those who were unvaccinated.

The higher absolute risk of VTE during the early waves can be attributed to several factors. There were significant disruptions to surgical services early in the pandemic, with an unprecedented number of operations being cancelled or delayed [18, 19]. As a result, numerous international guidelines were published advocating that elective surgical priority be given to the most urgent and severe cases [20]. Patients in these categories would have undoubtedly had higher baseline risks for VTE due to greater disease complexity and underlying comorbidity [21, 22, 23]. The higher virulence and transmissibility associated with the early SARS‐CoV‐2 variants, coupled with the limited availability of vaccines, likely contributed further to the increased risk during this period [24]. Between the Alpha and Delta waves of the pandemic, we showed a decline in the absolute risk of 90‐day VTE from 1.0% to 0.8%. The beginning of the Alpha wave coincided with the nationwide rollout of the vaccination programme for COVID‐19 in the UK. This timing is crucial and likely reflects the broader population‐level impact of vaccinations in reducing virus circulation and subsequent incidence of SARS‐CoV‐2 infections. In addition, the lessons learnt from previous waves led to the refinement of surgical pathways and enhanced peri‐operative care, improving patient outcomes and mitigating the risk of postoperative VTE [25].

Our study has shown a stepwise reduction in the risk of postoperative VTE following vaccination when adjusting for other confounders. This underlines the importance of vaccination in the prevention of VTE in those with peri‐operative SARS‐CoV‐2 infection. This supports the modelling work estimating that 60,000 deaths a year could be saved by widespread vaccination before surgery [26]. A study of 457,804 patients from the N3C Data Enclave in the USA reported a protective effect of vaccination on major cardiovascular events, and concluded that vaccination status and the timing of SARS‐CoV‐2 infection must form part of VTE risk stratification [27]. A smaller study using data from the Veterans Affairs Facilities involving 30,681 patients, of whom 3104 were vaccinated, reported an increased risk of thrombotic complications but was unable to report on VTE rates alone [28]. These studies have not been able to report rates beyond 30 days or across surgical specialties whilst accounting for important confounders. A recent systematic review reported higher rates of VTE during the pandemic in patients with SARS‐CoV‐2 infection; however, it highlighted the need for large population‐based studies that could account for multiple confounders [8].

Our results suggest that vaccination is protective against postoperative VTE in those who develop peri‐operative SARS‐CoV‐2 infection. This is important as it shows the beneficial effect of vaccination in patients undergoing surgery and, due to current restrictions in access to vaccinations, this may disadvantage some patients who do not qualify for repeated doses. Further work will be required to assess the longevity of protection from vaccination and the need for further booster vaccines.

One of the strengths of this study is the large population‐based cohort. The OpenSAFELY‐TPP platform allowed us to access linked primary and secondary healthcare records of over 1.8 million surgical procedures across England, including validated patient‐level data on SARS‐CoV‐2 infection and vaccination status alongside VTE event data. Access to surgical procedure timings in relation to the four waves of the pandemic also allowed for a more nuanced understanding of how these factors influenced postoperative risk during the pandemic. The size of the study has allowed us to consider a range of confounding variables that may have influenced the association between SARS‐CoV‐2 infection and postoperative VTE.

This study has some limitations. The use of electronic healthcare records may introduce misclassification bias due to human error during the coding process; however, this is likely to be minimal as NHS coding of procedures and diagnoses is standardised. We have used the admission date as the date of surgery because the exact date was not available. However, the admission day will be the same as the day of surgery in the majority of elective surgical procedures, as it is not standard practice to admit patients the day before surgery. Furthermore, during the pandemic there were additional attempts to limit time in hospital for patients undergoing elective procedures.

Although our models adjusted for many confounding factors, there may exist residual confounding by unmeasured variables such as local variations in inpatient thromboprophylaxis regimes or differences in surgical pathways and protocols. Another limitation is that our study combined a variety of surgical procedures and specialties into broad categories, which may have masked procedure‐specific risks and outcomes. Our study includes data until October 2022, so further work is required to detail the recovery phase from the pandemic. However, this study represents the largest and most contemporary study of its kind detailing VTE risk during this time period and the impact of vaccinations. Lastly, it is worth noting that the evolving nature of the pandemic meant that there were likely variations in viral strains, public healthcare responses and vaccination uptake across different regions in England that may have impacted patient outcomes and which could not be modelled fully in this study.

SARS‐CoV‐2 infection status and vaccination history had a substantial influence on 90‐day VTE risk, with a recent and a postoperative SARS‐CoV‐2 infection both conferring an increased risk, whilst one dose of vaccine reduced the risk. Using a large population‐based cohort, we have shown that those with a recent SARS‐CoV‐2 infection are still at an increased risk of VTE following surgery and so current guidance to delay surgery may be reasonable where clinical urgency and risk assessment allow. In addition, we have shown that those patients with a postoperative SARS‐CoV‐2 infection are also at increased risk, and this group may therefore benefit from extended thromboprophylaxis following surgery. This would require an expansion of current guidelines on who is at an increased risk of postoperative VTE and thus needs to be given prolonged prophylaxis [29].

## Supporting information


**Appendix S1.** Full acknowledgements.
